# The ant *Lasius niger* is a new source of bacterial enzymes with biotechnological potential for bleaching dye

**DOI:** 10.1038/s41598-019-51669-w

**Published:** 2019-10-23

**Authors:** Alexandra Díez-Méndez, Paula García-Fraile, Francisco Solano, Raúl Rivas

**Affiliations:** 1 Department of Microbiology and Genetics, University of Salamanca, Plaza Doctores de la Reina s/n, 37007 Salamanca, Spain; 2 Spanish-Portuguese Institute for Agricultural Research (CIALE), Salamanca, Spain; 3 Institute of Microbiology, Academy of Sciences of the Czech Republic, Videnska 1083, 142 20 Praha 4, Prague, Czech Republic; 4 Department of Biochemistry and Molecular Biology B. Faculty of Medicine and LAIB-IMIB, University of Murcia, 30100 Murcia, Spain; 5 Associated Unit USAL-CSIC (IRNASA), Salamanca, Spain

**Keywords:** Environmental sciences, Pollution remediation, Environmental impact

## Abstract

Industrial synthetic dyes cause health and environmental problems. This work describes the isolation of 84 bacterial strains from the midgut of the *Lasius niger* ant and the evaluation of their potential application in dye bioremediation. Strains were identified and classified as judged by rRNA 16S. The most abundant isolates were found to belong to Actinobacteria (49%) and Firmicutes (47.2%). We analyzed the content in laccase, azoreductase and peroxidase activities and their ability to degrade three known dyes (azo, thiazine and anthraquinone) with different chemical structures. Strain Ln26 (identified as *Brevibacterium permense*) strongly decolorized the three dyes tested at different conditions. Strain Ln78 (*Streptomyces ambofaciens)* exhibited a high level of activity in the presence of Toluidine Blue (TB). It was determined that 8.5 was the optimal pH for these two strains, the optimal temperature conditions ranged between 22 and 37 °C, and acidic pHs and temperatures around 50 °C caused enzyme inactivation. Finally, the genome of the most promising candidate (Ln26, approximately 4.2 Mb in size) was sequenced. Genes coding for two DyP-type peroxidases, one laccase and one azoreductase were identified and account for the ability of this strain to effectively oxidize a variety of dyes with different chemical structures.

## Introduction

Synthetic dyes are widely used in different types of production including the pharmaceutical, cosmetic, hair coloring, food coloring supplements, carpet and leather manufacturing and printing industries^1^. Consequently, the wastewater generated by these processes often contains a variety of chemicals that can seriously pollute the environment^2^. Industrial effluents can often contain quantities of reactive synthetic dyes ranging from 2% to 50% (w/v)^3^. However, copper-enzymes can catalyze the oxidation of different natural and xenobiotic compounds, such as phenols, polyphenols, amines, substituted polycyclic aromatic hydrocarbons, pesticides and synthetic dyes, by using atmospheric oxygen. Among these dyes, thiazine, azo and anthraquinone are the organic groups most commonly found, and all are recalcitrant and toxic to both the environment and humans in varying degrees^4^. Therefore, eliminating these compounds is essential in order to protect living beings and the natural world. Although different chemical methods have been proposed to achieve this goal, many are expensive and generate the problem of disposing the resulting sludge^5^. In recent years, there has been a trend toward developing eco-friendly solutions for degrading dyes based on bioremediation strategies. One of the best options for bioremediation is the use of microorganisms that contain oxidoreductases capable of degrading these types of dyes. Moreover, it is known that laccases, azoreductases and peroxidases are three types of enzymes frequently involved in these kinds of oxidative processes.

Among the first and most frequently used enzymes are laccases (E.C. 1.10.3.2), since they have the well-known ability to degrade resistant organic materials such as lignin^6,7^. As a result, laccases have received much attention, and in particular fungal laccases^8^, owing to their potential application in biotechnological processes. Furthermore, ever since laccase-like activity was initially detected in the bacteria *Azospirillum lipoferum*, the use of prokaryotic cells expressing these enzymes has increased due to their potential applications^8–12^.

Azoreductases (E.C 1.7.1.6) are enzymes that exist in a number of fungi and bacteria that allow these microorganisms to use azo dyes as an alternative carbon source^13^. Azoreductases are flavoenzymes that require a redox cofactor for catalyzing the degradative reactions, typically NAD(P)H^14^. These enzymes are also used to degrade azo dyes^15^, because they are able to cleave azo bonds producing simple amines and other decolourized aromatic compounds. In recent years, several bacterial azoreductases have been discovered, such as *Kocuria rosea, Bacillus subtilis, Xenophilus azovorans, Enterococcus faecalis* and *Shewanella S12*^16–20^, and used for similar types of purposes.

Peroxidases (E.C. 1.11.1.x) form the third group of enzymes that can be potentially used for dye degradation. These enzymes catalyze the oxidation of a number of compounds using hydrogen peroxide as the substrate^21,22^. Furthermore, the use of peroxides to remove azo dyes and other xenobiotic compounds from textile effluents is well established^23^. These enzymes are presented in both eukaryotic and prokaryotic organisms^15^ and new microbial peroxidases have also been recently described^24^.

Although fungi are the most commonly used microorganisms in the treatment of azo dyes, the use of bacteria has been increased in recent years as a way to improve the end result of the oxidative processes. Prokaryotic cells are able to carry out dye degradation faster and with wider specificity^25^. In addition, dye decolorizing bacteria have been isolated from different niches including soil, water, the animal gut, and even the human intestine^26,27^. Some reports indicate that there are other easy-to-handle and promising ecological niches, such as insects, that are practically unexplored. Preliminary studies have indicated that bacteria from the termite gut contain putative laccases involved in the oxidation of polyphenols and other related compounds present in plant biomass^28^. Bacterial strains isolated from the ant midgut have also been described in diverse species of the genera *Camponotus* and *Cephalotes*, suggesting oxidative degrading enzymes contribute to the digestion of xenobiotics ingested by these insects^29,30^. Furthermore, a beneficial symbiosis between the actinomycetes inhabiting the surface of black garden ants (*Lasius niger*) and their host has also been reported^31^. However, there are no studies related to the detailed nature of the living microbiota in ants and their potential application in dye degradation and bioremediation processes.

The present work describes the isolation and identification of a number of bacterial strains from *Lasius niger* and the evaluation of their potential application in bioremediation. We explored the nature of the oxidative enzymes contained in the strains identified that were found to effectively degrade three common dyes with different chemical structures. The correlations among oxidoreductases and the sort of dye preferentially degraded are discussed, and those oxidoreductases have been partially characterized. Data on the optimal pH and temperature for the two most active strains Ln26 and Ln78 have been presented and a complete genome sequence analysis of the strain Ln26 genome has been carried out to establish possible correlations between the genes identified and enzyme activities. The advantage and novelty of the present case is the use of ant microbiota as source of enzymes for bioremediation. We were looking for strains with wide specificity containing several complementary oxidoreductases acting on dyes of different chemical structure.

## Results

A total of 15 black garden ants (*Lasius* niger) were collected aseptically from soil in Calvarrasa de Arriba (N40°54′24.19″ O5°35′31.16″), Salamanca, Spain and subjected to surface-sterilization and crushing treatments. After a week of incubation on agar plates, a total of 93 microorganisms were isolated, of which 84 were bacterial strains and 9 were eukaryotes (4 filamentous fungi and 5 yeasts). Only the bacterial isolates were selected for this study and identified through sequencing the 16S rRNA gene. Fifty-five of these strains showed an overall similarity higher than 99% between their rRNA sequences and those of strains registered in the databases, and the strains were correctly identified and classified (Table 1). Isolates belonging to Actinobacteria were the most abundant (49%), followed by Firmicutes (47.2%) and Alphaproteobacteria (3.8%). The 27 isolates of the phylum Actinobacteria belonged to the genus *Streptomyces* (11), *Brevibacterium* (6), *Micrococcus* (6), *Rhodococcus* (2), *Micromonspora* (1) and *Dermacoccus* (1). There were also 26 isolates from the phylum Firmicutes, which was represented by the genera *Bacillus* (20), *Paenibacillus* (4), *Staphylococcus* (2). Two isolates from the phylum Alphaproteobacteria were isolated, represented by the genera *Roseomonas* (1) and *Sphingomona*s (1). The largest group, in terms of the number of species recovered, was the genus *Streptomyces* (5 species), followed by *Bacillus*, *Brevibacterium* and *Staphylococcus* (2), and *Roseomonas*, *Micromonospora*, *Dermacoccus*, *Paenibacillus*, *Sphingomonas* and *Micrococcus* (1).

**Table 1:** Analysis and identification of bacterial isolates from Lasius niger midgut based on 16S rRNA gene sequences. Threshold for identification was similarity greater than 99%. Table includes the closest match using NCBI databases.

Phylogenetic group	Bacterial isolate	Top match	Nucleotide identity (%)
Firmicutes	Ln01	*Bacillus aryabhattai* B8 + 22(T)	100
Proteobacteria	Ln06	*Sphingomonas paucimobilis* ATCC29837 (T)	100
Actinobacteria	Ln07	*Micrococcus luteus* YIM 65004 (T)	99.8
Firmicutes	Ln08	*Bacillus aryabhattai* B8 + 22(T)	100
Firmicutes	Ln09	*Bacillus aryabhattai* B8 + 22(T)	99.90
Firmicutes	Ln10	*Bacillus aryabhattai* B8 + 22(T)	100
Actinobacteria	Ln12	*Micrococcus luteus*YIM 65004 (T)	99.4
Actinobacteria	Ln13	*Micrococcus luteus* YIM 65004 (T)	99.7
Firmicutes	Ln14	*Paenibacillus cineris LMG* 18439 (T)	100
Firmicutes	Ln15	*Bacillus aryabhattai* B8 + 22(T)	99.7
Firmicutes	Ln16	*Bacillus aryabhattai* B8 + 22(T)	99
Firmicutes	Ln17	*Paenibacillus cineris LMG* 18439 (T)	99.4
Firmicutes	Ln18	*Paenibacillus cineris LMG* 18439 (T)	99.4
Firmicutes	Ln19	*Bacillus aryabhattai* B8 + 22(T)	99.6
Actinobacteria	Ln23	*Brevibacterium permense* VKMAc-2280(T)	99.1
Actinobacteria	Ln24	*Brevibacterium siliguriense* MB18 (T)	99.9
Actinobacteria	Ln25	*Brevibacterium permense* VKMAc-2280(T)	99.7
Actinobacteria	Ln26	*Brevibacterium permense* VKMAc-2280(T)	99.2
Actinobacteria	Ln27	*Brevibacterium siliguriense* MB18(T)	99.1
Firmicutes	Ln30	*Bacillus megaterium* NBRC15308 (T)	100
Actinobacteria	Ln34	*Brevibacterium siliguriense* MB18(T)	100
Firmicutes	Ln36	*Staphylococcus gallinarum* ATCC35539(T)	99.7
Firmicutes	Ln37	*Bacillus aryabhattai* B8 + 22(T)	100
Firmicutes	Ln39	*Bacillus aryabhattai* B8 + 22(T)	100
Firmicutes	Ln40	*Bacillus aryabhattai* B8 + 22(T)	100
Firmicutes	Ln41	*Bacillus aryabhattai* B8 + 22(T)	100
Firmicutes	Ln49	*Bacillus aryabhattai* B8 + 22(T)	99.8
Firmicutes	Ln50	*Bacillus aryabhattai* B8 + 22(T)	99.5
Firmicutes	Ln51	*Paenibacillus cineris LMG* 18439 (T)	99.3
Actinobacteria	Ln52	*Rhodococcus fascians* LMG 3623(T)	100
Actinobacteria	Ln53	*Rhodococcus fascians* LMG 3623(T)	99.4
Actinobacteria	Ln57	*Dermacoccus nishinomiyaensis* DSM 20448(T)	99.2
Proteobacteria	Ln59	*Roseomonas mucosa* ATCC-BAA-692 (T)	100
Firmicutes	Ln60	*Bacillus aryabhattai* B8 + 22(T)	99.9
Firmicutes	Ln61	*Bacillus aryabhattai* B8 + 22(T)	99.7
Actinobacteria	Ln62	*Streptomyces ambofaciens* ATCC 23877 (T)	99.0
Actinobacteria	Ln63	*Streptomyces thioluteus* LMG20253(T)	100
Actinobacteria	Ln66	*Micrococcus luteus /yunennesis* YIM 65004 (T)	100
Actinobacteria	Ln67	*Micrococcus luteus /yunennesis* YIM 65004 (T)	99.9
Actinobacteria	Ln69	*Micrococcus luteus /yunennesis* YIM 65004 (T)	99.7
Actinobacteria	Ln74	*Micromonospora zamorensis* CR38(T)	100
Actinobacteria	Ln75	*Streptomyces ambofaciens* ATCC 23877 (T)	99.6
Actinobacteria	Ln76	*Streptomyces violaceochromogenes* NBRC 131100 (T)	99.7
Actinobacteria	Ln77	*Streptomyces pactum* NBRC 13433(T)	100
Actinobacteria	Ln78	*Streptomyces ambofaciens* ATCC 23877 (T)	99.8
Actinobacteria	Ln79	*Streptomyces ambofaciens* ATCC 23877 (T)	99.6
Actinobacteria	Ln80	*Streptomyces iakyrus* NBRC 13401 (T)	99.7
Actinobacteria	Ln81	*Streptomyces iakyrus* NBRC 13401 (T)	99.7
Actinobacteria	Ln82	*Streptomyces violaceochromogenes* NBRC 131100 (T)	99.7
Actinobacteria	Ln83	*Streptomyces ambofaciens* ATCC 23877 (T)	100
Firmicutes	Ln84	*Bacillus aryabhattai* B8 + 22(T)	100
Firmicutes	Ln87	*Bacillus aryabhattai* B8 + 22(T)	100
Firmicutes	Ln88	*Bacillus aryabhattai* B8 + 22(T)	100
Firmicutes	Ln90	*Bacillus aryabhattai* B8 + 22(T)	100
Firmicutes	Ln93	*Staphylococcus sciuri subsp. sciuri* DSM20345(T)	100

### Decoloration plate assay and strains with the ability to decolor dyes

Biodegradation of the synthetic dyes Congo Red (CR), Toluidine Blue (TB) and Remazol Brilliant Blue (RBB) by the bacterial strains under aerobic conditions was studied using a decoloration assay in agar plates. The assay conditions were laccase-like (no extra additions), azoreductase-like (addition of NADH as enzyme cofactor) and peroxidase-like (addition of H_2_O_2_ as enzymatic substrate). The bacterial strains exhibited varying abilities to generate decolored halos on plates containing CR, TB and RBB. Forty-one strains were able to oxidize at least one of the three dyes. The decoloration activity was expressed in qualitative terms and classified as weak (W), positive (+) and strong positive (++), according to the size and time of appearance of a decolored halo (Table 2).

**Table 2:** Dye decoloration and DMP oxidation (column on the right) capabilities of bacterial strains isolated from *Lasius niger* midgut ant showing some decoloration effect in at least one condition. CR: Congo Red; TB: Toluidine Blue; RBB: Remazol Brilliant Blue R. Degree of decoloration after 1 week is classified as negative (−), weak positive (W), positive (+) and strong positive (++).

Bacterial strain	Control media (Laccase-like)			NADH addition (Azoreductase-like)			H_2_O_2_ addition (Peroxidase-like)			Control
	CR	TB	RBB	CR	TB	RBB	CR	TB	RBB	DMP
Ln01 *Bacillus aryabhattai*	−	**+**	−	−	**+**	−	−	−	−	**+**
Ln06 *Sphingomonas paucimobilis*	**+**	−	**+**	−	**+**	**+**	**+**	−	**+**	**+**
Ln08 *Bacillus aryabhattai*	−	**+**	−	−	−	**W**	−	−	**+**	−
Ln09 *Bacillus aryabhattai*	**+**	−	−	**W**	**+**	**W**	−	−	**+**	**+**
Ln10 *Bacillus aryabhattai*	−	**+**	−	−	**+**	**W**	−	−	−	**+**
Ln12 *Micrococcus luteus*	**+**	−	**W**	**W**	−	−	**+**	−	−	**+**
Ln13 *Micrococcus luteus*	**+**	**+**	**W**	**W**	−	−	−	−	−	−
Ln14 *Paenibacillus cineris*	**+**	**+**	**W**	−	**+**	**++**	−	−	**W**	**+**
Ln15 *Bacillus aryabhattai*	**+**	**+**	−	−	**W**	−	−	−	−	**+**
Ln16 *Bacillus aryabhattai*	−	−	−	−	−	−	**+**	−	−	−
Ln17 *Paenibacillus cineris*	**+**		**W**	−	−	−	−	−	−	**+**
Ln18 *Paenibacillus cineris*	−	**+**	**+**	−	**+**	−	−	−	−	**+**
**Ln26** ***Brevibacterium permense***	++	++	**W**	+	++	**W**	−	++	**W**	+
Ln27 *Brevibacterium siliguriense*	+	−	−	−	−	−	−	−	**W**	**+**
Ln30 *Bacillus megaterium*	−	−	−	**+**	−	−	−	−	−	**+**
Ln34 *Brevibacterium siliguriense*	**W**	−	−	−	−	−	−	−	−	**+**
Ln36 *Staphylococcus gallinarum*	**W**	−	**W**	−	**+**	−	−	−	−	**+**
Ln37 *Bacillus aryabhattai*	**+**	**+**	**W**	−	**+**	**+**	−	−	−	**+**
Ln39 *Bacillus aryabhattai*	−	−	−	−	**+**	**+**	−	−	−	**+**
Ln40 *Bacillus aryabhattai*	−	−	−	−	−	−	−	−	**W**	−
Ln41 *Bacillus aryabhattai*	−	−	−	−	**+**	**+**	−	−	**W**	**+**
Ln49 *Bacillus aryabhattai*	−	−	**+**	−	−	−	−	−	−	**+**
Ln52 *Rhodococcus fascians*	**+**	**W**	−	−	**+**	−	−	−	**W**	**+**
Ln53 *Rhodococcus fascians*		**W**	−	**+**	**+**	−	**W**	−	**W**	**+**
Ln57 *Dermacoccus nishinomiyaensis*	**+**	−	**+**	**+**	**+**	**+**	**+**	**W**	−	**+**
Ln59 *Roseomonas mucosa*	−	**W**	−	−	**+**	−	**W**	**W**	−	**+**
Ln61 *Bacillus aryabhattai*	−	−	−	−	**+**	−	−	−	**+**	
Ln62 *Streptomyces ambofaciens*	−	−	−	−	−	−	−	−	**+**	**+**
Ln63 *Streptomyces thioluteus*	−	−	−	−	−	**+**	−	−	**+**	
Ln66 *Micrococcus luteus /yunennesis*	**+**	−	−	**W**	−	−	−	−	−	**+**
Ln67 *Micrococcus luteus /yunennesis*	**+**	**+**	−	−	−	−	**+**	**W**	−	**+**
Ln69 *Micrococcus luteus /yunennesis*	−	−	−	−	−	−	−	−	**W**	−
Ln74 *Micromonospora zamorensis*	−	−	−	−	−	−	−	−	**W**	−
**Ln78** ***Streptomyces ambofaciens***	−	**++**	−	−	**++**	−	−	**++**	−	**+**
Ln80 *Streptomyces iakyrus*	−	−	−	−	−	−	**+**	−	−	**+**
Ln82 *Streptomyces violaceochromogenes*	−	−	−	−	−	−	−	−	**W**	**+**
Ln84 *Bacillus aryabhattai*	**+**	−	**+**	−	−	**+**	−	−	−	**+**
Ln87 *Bacillus aryabhattai*	−	**+**	−	−	**++**	−	−	−	−	**+**
Ln88 *Bacillus aryabhattai*	−	**+**	−	−	**++**	**+**	−	−	−	
Ln90 *Bacillus aryabhattai*	**+**	**+**	−	−	**+**	**+**	−	−	−	**+**
Ln93 *Staphylococcus sciuri subsp. sciuri*	**+**	−	−	−	−	−	−	−	−	**+**
Number of strains (+)/number of weak (W)	19/2	17/3	12/7	8/4	21/1	13/3	8/2	5/3	16/10	**32**

Comparison of Tables 1 and 2 indicates that 14 of the identified bacterial strains (Ln07, Ln19, Ln23, Ln24, Ln25, Ln50, Ln51, Ln60, Ln75, Ln76, Ln77, Ln79, Ln81 y Ln83) were unable to decolorize any of the dyes under any of the conditions. This indicated that these strains did not express the enzymes assayed and were therefore discarded.

Interestingly, the degradation of each dye showed a certain correlation with some of the three conditions assayed. CR was decolorized by 19 strains under laccase-like conditions, but only 8 strains were able to act on this azo dye in the presence of NADH or H_2_O_2_. TB was preferentially decolorized under azoreductase-like conditions by 21 strains, and RBB was preferentially decolorized in the presence of H_2_O_2_ (peroxidase-like conditions) by 16 strains.

Some strains showed specific activity, where Ln16, Ln30, Ln49 and Ln93 only showed activity in the presence of CR under the three different conditions. In turn, Ln49 only degraded RBB under laccase-like conditions and strains Ln39 and Ln41 were capable of degrading TB and RBB, but only in the presence of the NADH cofactor. On the other hand, the addition of NADH or H_2_O_2_ enhanced the decolorizing potential of some strains in comparison to laccase-like conditions but decreased the action of the others. Although these specific features are interesting regarding the nature of the enzymes contained by the different strains, the application of these differences in bioremediation processes is not clear yet.

On the other hand, Table 2 also shows the unspecific oxidation capacity of these strains on DMP (right column). DMP is a chromogen that can be used for the easy detection of oxidizing activity. According to the results, a high number of strains tested positive (32 out of 41).

### Decoloration assay of Ln26 in liquid medium

**Figure 1:**
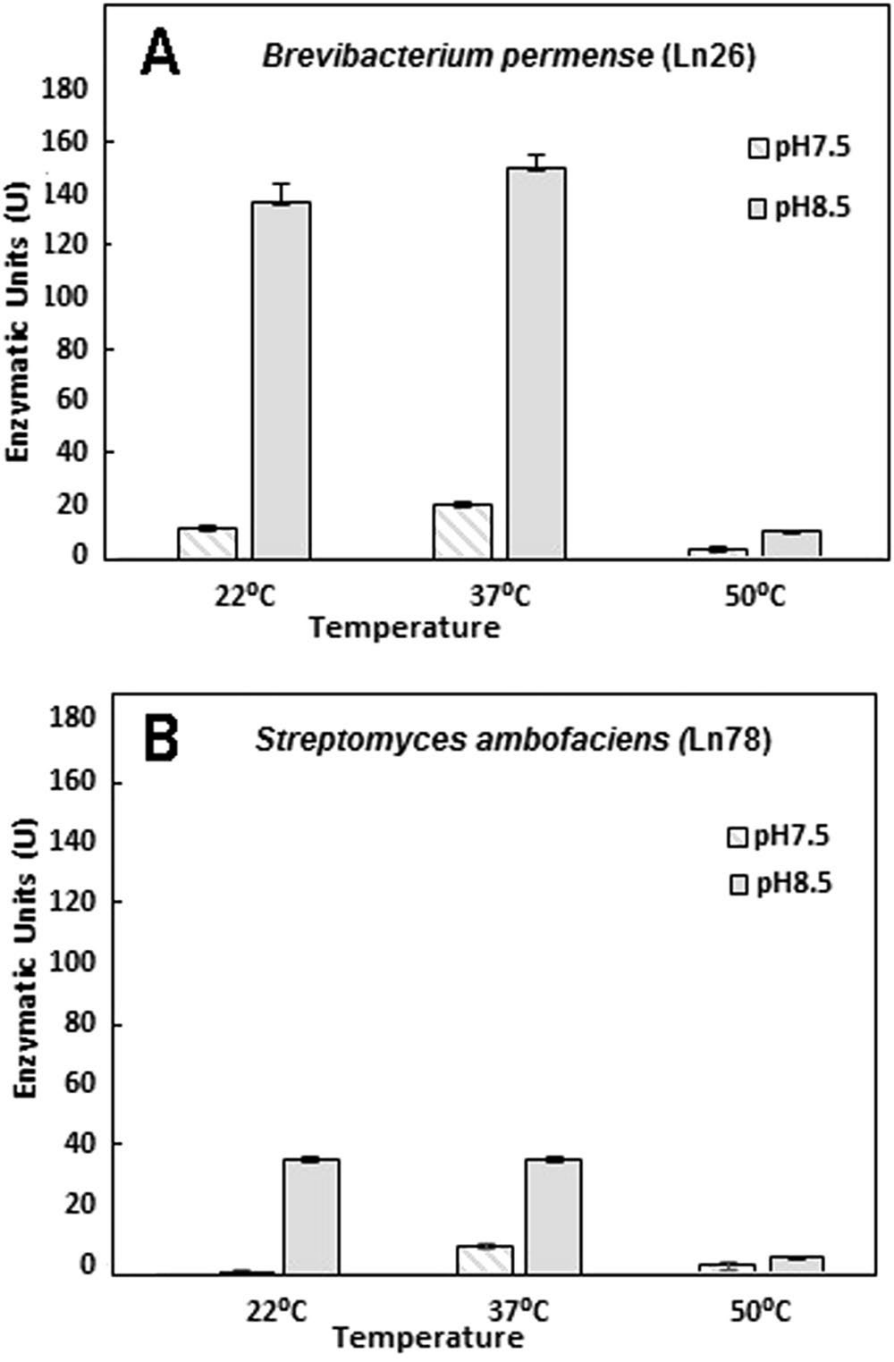
DMP oxidase activity of the bacterial strains Ln26 (**A**) y Ln78 (**B**) at selected pHs (7, 5 and 8.5) and temperatures (22, 37 and 50 °C). Activities are expressed as enzymatic units in comparison to a commercial laccase.

Remarkably, Ln26 produced the strongest and most versatile decoloration activity on the three synthetic dyes under several conditions, making it the best candidate for future use in bioremediation. This strain was identified as *Brevibacterium permense* (Tables 1 and 2). Thus, the decoloration capacity of the Ln26 strain was also tested in liquid medium under identical conditions that the agar plate assay by without agar addition. The three dyes were decolorized, although RBB was more resistant than RC and TB (Figure 1S), in accordance with the results obtained in the solid medium (Table 2). The residual % of dyes after 88 h was 44, 62 and 64% for TB, RC and RBB respectively.

### Effect of temperature and pH on the activity of extracts taken from selected strains

In addition to Ln26, Ln78 (identified as *Streptomyces ambofaciens*), showed strong oxidizing activity for TB degradation, irrespective of the absence or presence of cofactors (Table 2). Thus, we selected Ln26 and Ln78 to determine the optimal temperature and pH conditions for expressing maximum activity, using DMP as substrate for this series of experiments. Concerning pH, no activity was observed in the citrate buffer at pH 5. Using the phosphate buffer, however, activity started to be detected at pHs greater than 6, which significantly increased at pHs greater than 7. Figure 1 shows the activities at pH 7.5 and 8.5. The latter was found to be the optimal pH for both strains, although, as expected, Ln26 exhibited higher oxidative ability than Ln78. Regarding temperature, the activities were very similar at 22 °C and 37 °C, but greatly decreased at 50 °C, suggesting that stability of the oxidizing enzymes is not maintained at that temperature.

### Genomic properties

The draft genome sequence of strain Ln26 consists of 102 contigs with a total combined contig size of 4,188,813 bp and an average GC content of 64.3%. One rRNA operon, 48 tRNAs, and 3,775 coding sequences (CDSs) were also found. Other features of the bacterial chromosome are summarized in Table 3. This genomic sequence has been deposited in GenBank under the accession number SAMN09982396.

**Table 3:** Main features of the draft genome sequence of *B. permense* Ln26.

Characteristics	*B. permense* (Ln26)
Genome size (bp)	4,188,813
GC content	64,3
Number of contigs	102
Predicted coding sequences	3775
Subsystems	413
Number of RNAs	51
Auxiliary activities(AA)	5
Carbohydrate-binding modules (CBM)	2
Carbohydrate esterases (CE)	16
Gycosyl transferases (GT)	34
Glysosyl hydrolases (GH)	15
Polysaccharide lyases (PL)	Not found

### Sequence data mining and search of genes encoding oxidizing enzymes

**Figure 2:**
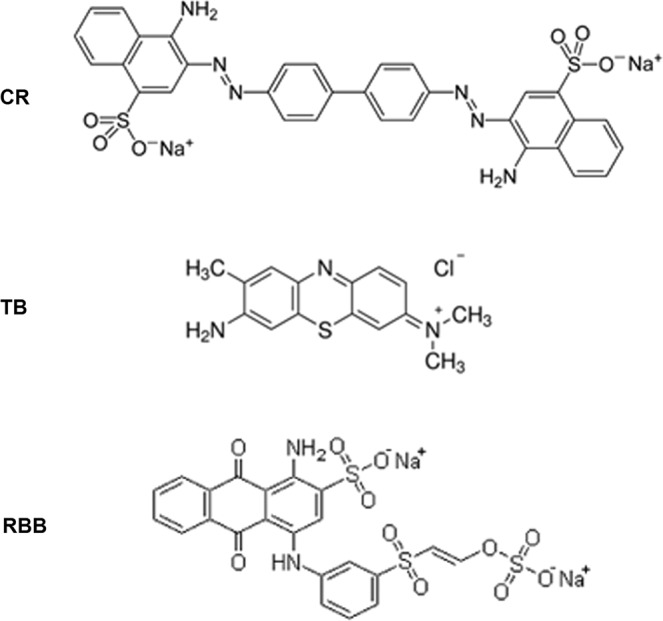
Chemical structure of the three model dyes used in this study. Red Congo (RC, azo dye); Toluidine Blue TB, thiazine dye) and Remazoll Brilliant Blue R (RBB, anthraquinone dye).

As strain Ln26 showed significant potential to be used in bioremediation, its genome was sequenced to evaluate the presence of oxidoreductase enzymes. The SEED viewer and BlastKOALA genomic tools were used for the search. It was found that the strain Ln26 contained a cluster of genes encoding DyP-type peroxidase enzymes. Additionally, oxidoreductase enzymes in genus *Brevibacterium* were searched at the NCBI protein database (https://www.ncbi.nlm.nih.gov/protein/). All sequences found were compared with the Ln26 *Brevibacterium permense* genome using Blastp^32^. Some genes were identified encoding possible laccase and azoreductase enzymes that had not been previously annotated. In parallel, Cu-oxidase, Dyp-type and FMN-dependent protein domains were found using Motif scan tool (https://myhits.isb-sib.ch/cgi-bin/motif_scan) and NCBI’s Conserved Domain Database (CDD) (https://www.ncbi.nlm.nih.gov/pmc/articles/PMC5210587/). All of these studies and searches are included in Supplementary Material (Figure 2S).

## Discussion

Two of the most important factors concerning the use of oxidative enzymes for dye decolorization in bioremediation processes are the type of oxidoreductase and the chemical structure of the dyes. A possible correlation between both factors would be significantly helpful for designing efficient systems.

In this study, we isolated and characterized a number of bacterial strains obtained from the ant midgut. In fact, ant microbiota is an easy and essentially unexplored source. The classification and identification of the strains was performed using 16S rRNA sequences. All bacterial isolates were classified into 11 genera. Three of these (*Staphylococcus*, *Enterococcus* and *Bacillus)* have previously been described as symbionts isolated from the midgut of fire ants^33,34^. The two other isolates (*Streptomyces* and *Dermacoccus)* have been described as being associated with leaf-cutting-ants^35^. Moreover, it has been reported that the genus *Streptomyces* can act as an ectosymbiont on the surface and nests of *Lasius niger*^31^, suggesting that *Streptomyces* is a common symbiont of ants and could play a role in their lifecycle. The remaining 6 genera (*Roseomonas*, *Sphingomonas, Micrococcus*, *Paenibacillus*, *Brevibacterium* and *Rhodococcus)* have never been previously described as being associated with any type of ants, including *Lasius niger*.

In turn, according to our results, some isolates may contain oxidoreductases. Briefly, laccases are multicopper oxidoreductases that show relatively wide specificity using atmospheric oxygen as the oxidant substrate^36^. Azoreductases are oxidoreductases that act on nitrogenous organic compounds using NAD(P)H as cofactors^14^. According to their name, most azoreductases are able to cleave azo bond in dyes containing this chemical group, although sulfonated dyes are resistant to degradation^37^. Peroxidases are powerful oxidoreductases, as they use hydrogen peroxide as co-substrate. We have chosen three standard dyes, with different chemical structures, widely used in textile and biotechnological processes. In total, three types of dyes were used: Red Congo (an azo dye, sulfonated, acidic and usually available as sodium salt); Blue Toluidine (a thiazine, basic, usually available as chloride); and Remazol Brilliant Blue R (an anthraquinone, sulfonated, acidic, usually as sodium salt).

We found a variety of bacterial strains with different abilities to decolorize the 3 dyes assayed. Laccases are the most suitable enzymes, since they do not require cofactors or co-substrate, but only atmospheric oxygen. Although it has been reported that sulfonated azo dyes, such as CR, are resistant to laccase activity^38^, we found that a significant number of strains (19) were able to degrade CR (laccase-like conditions, Table 2). By contrast, we also found some strains, such as Ln39 and Ln41, which were unable to decolourize CR in the absence of cofactors but were able to degrade CR in the presence of NADH. This pattern suggests the expression of azoreductases in these strains. It has been reported that certain azoreductases are able to reduce sulfonated azo dyes such as the enzyme produced by *Bacillus sp*. B29^39^.

The number of strains that show activity on Red Congo in the presence of NADH is similar to the number of strains that show activity in the absence of NADH (laccase-like conditions) (19 vs 17). In sum, the addition of NADH generally does not improve the efficiency of this sulfonated azo dye, suggesting that the action of active bacterial strains is mostly due to laccases rather than to azoreductases.

According to previous reports^17^ and the results presented here, azoreductases are capable of decolorizing TB. Most of our strains effectively degrade TB in the presence of NADH (21), but some strains are also active in the absence of this cofactor. This point out that both laccases and azoreductases are able to act on basic thiazine dyes.

As far as we know, the effect of laccases and azoreductases on RBB and related sulfonated anthraquinones is not described in the literature^40^. Our results indicate that RBB is mostly degraded in the presence of H_2_O_2_ (peroxidase-like conditions). Sixteen strains are able to decolorize this dye under these conditions, but only 8 are capable of catalyzing under laccase-like conditions, and only 5 under azoreductase-like conditions. However, most of the strains active in the presence of H_2_O_2_ presented weak activity (10 out 16), indicating that this dye is not easily decolorized even by bacterial strains presumably containing peroxidase-like enzymes.

In addition to the classification of these types of enzymes, the use of efficient strains with wide specificity in industrial processes, together with simple and cheap conditions, is particularly interesting. Laccases seem to be the most efficient and inexpensive enzymes for dye decoloration, as they do not require the addition of NADH or hydrogen peroxide. The addition of these molecules to environments inoculated with bacteria for the purpose of bioremediation would enhance the contribution of azoreductases or peroxidases, but this would however be more difficult to carry out and more costly.

The optimization of conditions for achieving higher levels of enzymatic activity is also important. DMP was used for those particular experiments, owing to its wide specificity as the substrate of oxidoreductases, laccases^41,42^, azoreductases^17,37,43^ and peroxidases^22,24^. Ln 26 was able to partially decolorize the three dyes in liquid medium after around 3,5 days, although RBB was the most resistant one (Figure 1S). Longer incubations could not followed due to artifacts in the cultures (excessive cell growth, pH changes affecting the color of the dye left and so on). Further variations might improve the yield of decoloration, but this approach would wait for possible future studies. We also studied the effect of pH and temperature in the activity of cellular extracts of the two selected strains: Ln26, which shows a wide specificity for dyes and Ln78 which is specific for TB (Table 2).

Figure 1 shows that basic pHs (8.5) and moderate temperatures up to 37 °C are the most efficient conditions for both strains, but acidic pHs or temperature of 50 °C are not compatible with enzymatic activities. Remarkably, the enzymatic activity was quite similar at 22 °C and 37 °C, so that possible applications using these strains could take place without warming the reaction media, which is an important factor for saving energy costs.

Ln26 has been identified as *Brevibacterium permense*, and this is the first time that this bacteria have been sequenced and deposited in Genebank database. Consequently, the genome of Ln26 was explored to identify possible oxidoreductases involved in the dye-bleaching assays detected throughout this study. According to our search and the subsequent selection of Ln26, the genus *Brevibacterium* has been reported as showing great potential with regard to the biodegradation of different industrial dyes. For instance, Dyp-type enzymes have been reported in *Brevibacterium linens* M18^44^, as well as tyrosinase activity in *Brevibacterium* sp. strain VN15^45^. The interest for these oxidizing enzymes in bioremediation processes is growing, as recently reviewed^46^. In addition, we found that the strain Ln26 contains genes encoding for two different Dyp-type peroxidases, one laccase and one azoreductase. To our knowledge, this is the first report describing the existence of one laccase and one azoreductase in this species. These data strongly suggest that the genus *Brevibacterium* could be a promising candidate when designing bioremediation processes to degrade different chemical dyes.

## Conclusion

The midgut of black garden ants is a useful source of bacterial strains containing oxidative enzymes capable of decolorizing synthetic recalcitrant dyes. As a result of the screening carried out in this work, we identified a strain specifically capable of degrading TB dye (Ln78) and a highly promising strain with wide specificity and the ability to decolor all type of dyes in different conditions (Ln26). Thus, the strain Ln26 seems to be the best candidate for using in bioremediation processes and, consequently, its genome was sequenced and genome mining was performed in order to examine the presence of genes encoding oxidoreductases. Genome sequencing is an effective tool for searching for correlations among the enzymatic activities measured and the occurrence of genes encoding different forms of oxidoreductases. Using both RAST and BlastKoala tools, we detected genes encoding the 3 different types of oxidoreductases previously described, confirming the potential use in bioremediation processes for degrading dyes. These three enzyme types appear to be commonly found in *Brevibacterium* genus, supporting the belief that this genus could be useful in bioremediation. Furthermore, as the optimal conditions for decolorizing a particular dye depend on the conditions and the addition of cofactors specific for azoreductase or peroxidase, our results indicate that DMP could be used as a wide non-specific assay for detecting general oxidizing activity. Nevertheless, laccase activity seems to be the most useful for bioremediation reactions, due to the fact that this enzyme does not need the addition of other co-substrates.

## Methods

### Bacterial isolation

All bacterial strains were isolated from the *Lasius niger* midgut. Initially, the trapped ants were disinfected using a 2.5% Cl_2_Hg solution for 10 min then washed five times with sterilized H_2_O. The disinfection was performed to ensure that ants did not contain any external microorganisms on their bodies. Afterwards, the ants were crushed and plated under aseptic conditions on Yeast Mannitol Agar (YMA)^47,48^ and Yeast Extract Dextrose medium (YEDP) (glucose 7 g/L; Bacto Yeast Extract 3 g/L; calcium hydrogen phosphate anhydrous 3 g/L and agar 15 g/L). All plates were incubated at 28 °C for 7 days. Colonies were selected based on colour, shape, and size and the individual colonies were re-streaked on fresh YMA or YEDP plates until a pure culture was obtained.

### DNA Extraction and 16S rRNA amplification and sequencing

DNA for amplifying and sequencing 16S rRNA was extracted from pure colonies using the REDExtract-n-Amp^TM^ Plant PCR Kit (Sigma) following the manufacturer’s instructions. First, amplification of the 16S rRNA was performed using bacterial DNA as the template, The primers 27F (5′AGAGTTTGATCCTGGCTCAG) and 1522R (5′AAGGAGGTGATCCANCCRCA) were used for the PCR reaction under the following conditions: preheating at 95 °C for 9 min, 35 cycles of denaturation at 95 °C for 1 min, annealing at 54 °C for 1 min and extension at 72 °C during 1 min. The final extension in the last cycle was performed at 72 °C for 7 min. The amplicons (approximately 1200 bp) were checked on a 1% (w/v) agarose gel and the GeneRuler 1 kilobase DNA Ladder (Thermo Scientific ^TM^) was used as the size marker. For purification of DNA fragments, GeneJet Gel Extraction and DNA Cleanup Micro Kit (Thermo Scientific ^TM^) was used according to the manufacturer’s instructions. The sequences obtained were assembled using BioEdit software^49^ to obtain contig sequences for each strain. The isolates were identified using NCBI’s Blastn (https://blast.ncbi.nlm.nih.gov/Blast.cgi?PAGE_TYPE=BlastSearch).

### Decoloration assays on solid and liquid media

Congo Red (CR), Toluidine Blue (TB) and Remazol Brilliant Blue R (RBB) were used as the test dyes for the detection of bacterial strains able to decolorize recalcitrant compounds. These dyes are widely used in industrial processes and were selected based on their different chemical structures: azo (CR), thiazine (TB) and anthraquinone (RBB) (Fig. 2). The ability of the bacterial strains to degrade dyes was confirmed by the presence of a decolorized halo around each colony after 7 days in a plate assay. Control conditions were appropriate for laccase-containing strains. Moreover, the effect of different cofactors such as NADH, H_2_O_2_ favouring azoreductase and peroxidase activities were also evaluated. All dyes were purchased from Sigma and were added to the media after autoclaving as sterile-filtered water solutions to avoid any chemical decomposition due to high temperature. Dye decoloration was carried out in agar plate assays using squared Petri dishes containing 50 ml of YE medium (Yeast Extract 4 g/L; Agar 15 g/L) supplemented with 0.05% (w/v) of the each dye^49^. In addition to the control series, lacking any additive that may have facilitated laccase-like activity, another series of plates were supplemented with either 0.1 mM NADH or 1 mM H_2_O_2_ as cofactors, in order to enhance the oxidative ability of bacterial strains containing azoreductase-like or peroxidase-like activities, respectively. Another set of plates, in addition to the plates containing the standard dyes, were supplemented with 1 g/L 2,6-dimethoxyphenol (DMP)^50^ as an auxiliary indicator of the oxidative ability of bacterial strains (see below). All agar plates were inoculated by adding 5 µl aliquots of bacterial solutions (around 10^7^ cells mL^−1^) and incubated at 28 °C for 7 days. The plates were monitored daily by visual inspection. Oxidizing activity was considered positive when a diffuse (weak activity) or clear halo (+) was observed around the colonies at the end of the 7th day, although in the most active strains, halos were already evident during the 1st-3rd days of incubation (marked as strong positive (++) at Table 2).

Quantitative estimation of dyes decoloration by Ln26 was also carried out in liquid media assay using laboratory flasks containing 30 mL of YE medium supplemented with 0,05%(w/v) of each dye. Other laboratory flask was supplemented with 1 g/L DMP used control of the oxidizing potential of the bacterial strain. All flasks were inoculated by adding 1 mL of a fresh bacterial sample (around 10^7^ cells mL^−1^). Then, flasks were incubated at 28 °C and aerated by gentle shaking at 180 rpm during 5 days. We used three replicates for each condition. At the beginning of the experiment (time zero) and subsequently at several periods of time, samples of 1 mL were pipetted out from the flasks and centrifuged at 10.000 rpm to settle down the bacterial pellet. The clear supernatants containing the residual decolorized dye were monitored by measuring the absorbances of the samples at wavelengths 468, 498, 590 and 590 nm for DMP, CR, TB, and RBB respectively. Some supernatants should be diluted 10-times before absorbance determination to get appropriate linear range between absorbance and concentration. According this approach, the monitoring was not possible longer than 4 days due to artifacts in the color of residual dyes related to pH changes and late phase of the bacterial growth.

### Enzyme extraction

Selected bacterial strains were inoculated in YE broth medium and the cultures were incubated for 24 h at 28 °C with gentle shaking at 180 rpm. Afterwards, each sample was centrifuged at 10,000 rpm for 10 minutes at 4 °C, the pellets were washed twice with sterilize H_2_O and centrifuged after each wash under the same conditions. Finally, cell disruption was done by sonication at a frequency of 20 kHz 5 pulses of 10 sec. Sonicated samples were incubated 5 min on ice and centrifuged at 12,000 rpm for 12 min at 4 °C. The supernatants were considered to contain the crude enzymatic extracts and were stored at −20 °C until using for searching optimal conditions of pH and temperature. Protein was determined using the Bradford assay.

### Effect of temperature and pH on DMP oxidation activity in selected strain extracts

Oxidative enzymatic activity under different of pH and temperatures conditions was determined using DMP as the chromogenic substrate^51^. This dimethoxyphenol is a typical colourless laccase substrate that is also easily oxidized to yellowish oligomers by other oxidative enzymes. The increase of absorbance was measured at 468 nm in a spectrophotometer Asys UVM340. Reactions were monitored in 96 well plates using solutions of 0.8 g/L DMP in 100 mM sodium 100 mM citrate buffer (for pH 5) or 100 mM sodium phosphate buffer (for pHs 6, 7.5 and 8.5). Aliquots of 20 µl from each tested bacterial strain extract (protein concentration were 2.2 and 3.1 mg/ml for Ln26 and Ln78 respectively) were added to 180 µl of DMP solution in the appropriate buffer and temperature (22 °C, 37 °C and 50 °C). Initial A_468_ were measured, and the plates were incubated overnight and A_468_ were again determined. Negative controls reactions were carried out under identical condition, but 20 µl of the appropriate buffer was added instead of the bacterial extracts. Commercial laccase from *Trametes versicolor* was purchased from Sigma and used as positive control. A calibration curve was plotted using amounts of the laccase ranging from 0.08 to 87 units to correlate A_468_ and units of laccase activity. According to the instructions provided by the manufacturer for laccase originating from *Trametes versicolor o*ne unit corresponds to the amount of enzyme that converts 1 µmole of catechol per minute at pH 5.0 and 25 °C. All experiments were performed in triplicate.

### Draft genome sequence, annotation and genotypic analysis of the strain Ln26

The genomic DNA for genome sequencing was obtained from bacterial cells of Ln26 strain grown on TSA plates and collected after 24 h at 28 °C, using the ZR Fungal/Bacterial DNA MiniPrep (Zymo Research). The draft genome sequence of the isolated was obtained by shotgun sequencing on an Illumina MiSeq platform via a paired-end run (2 × 251 bp). Sequence data was assembled using Velvet 1.2.10^52^. Gene calling and annotation was performed using RAST 2.0 (Rapid Annotation using Subsystem Technology)^53^ and The SEED viewer framework^54^ and BlastKOALA tools^55^ were used to search for genes related to dye decoloration.

### Nagoya protocol

All the bacterial strains used in this study were isolated before the signed of the Nagoya Protocol.

## Supplementary information

Figure 1S

Figure 2S
