# Supplementary material for: The ant Lasius niger is a new source of bacterial enzymes with biotechnological potential for bleaching dye
Source: Sci Rep. 2019 Oct 23;9:15217. doi: 10.1038/s41598-019-51669-w (PMC6811527; doi:10.1038/s41598-019-51669-w)

**The ant *Lasius niger* is a new source of bacterial enzymes with biotechnological potential for bleaching dye.**

Díez-Méndez, Alexandra^1,2*^, García-Fraile, Paula^1,2,3^ Solano, Francisco^4^ and Rivas, Raúl.^1,2,5^


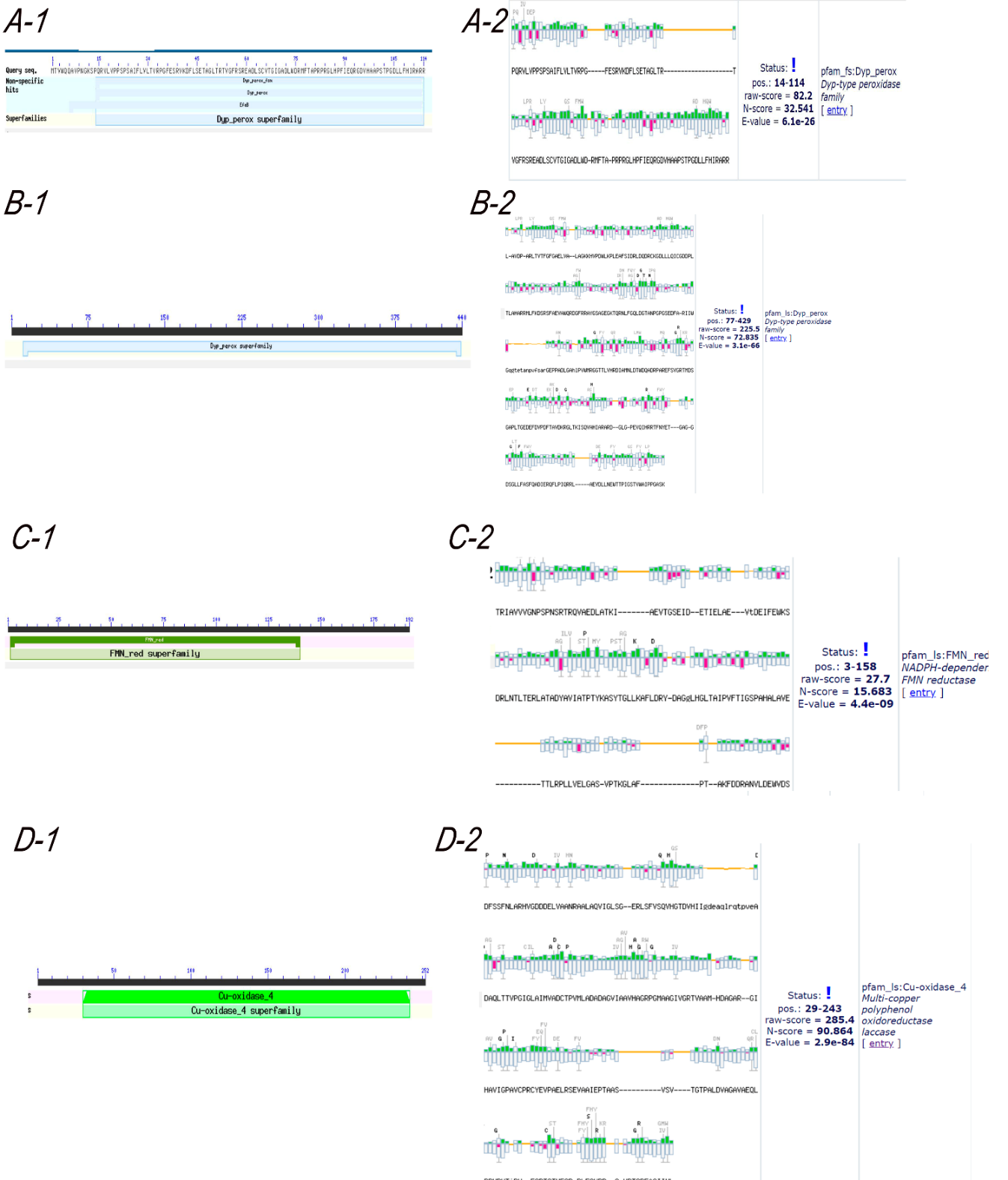

Supplement: Supplementary file 2 — Figure 2S [file 41598_2019_51669_MOESM2_ESM.docx]
